# Rapid Production of a Novel Al(III) Dependent Bioflocculant Isolated From *Raoultella ornithinolytica* 160-1 and Its Application Combined With Inorganic Salts

**DOI:** 10.3389/fmicb.2020.622365

**Published:** 2021-01-12

**Authors:** Rui Ding, Laipeng Luo, Ruixiang Han, Meiling Zhang, Tingting Li, Jihui Tang, Shenghai Huang, Jiong Hong

**Affiliations:** ^1^School of Life Sciences, Anhui Medical University, Hefei, China; ^2^Laboratory Department of Anhui Medical University, Hefei, China; ^3^School of Pharmacy, Anhui Medical University, Hefei, China; ^4^School of Life Sciences, University of Science and Technology of China, Hefei, China; ^5^Hefei National Laboratory for Physical Science at the Microscale, Hefei, China

**Keywords:** *Raoultella ornithinolytica*, bioflocculant, inorganic flocculant, composite bioflocculants, flocculation mechanism

## Abstract

An efficient bioflocculant-producing strain, *Raoultella ornithinolytica* 160-1, was identified by 16S rRNA and mass spectrometry analyses. Rapid production of bioflocculant EPS-160 was obtained with 10.01 g/(L⋅d) after optimized by response surface methodology. With the aid of Al(III), more than 90% flocculation activity of EPS-160 at 8 mg/L dosage was achieved in 5 min. Thus, this novel Al(III) dependent bioflocculant was used in combined with chemical coagulants AlCl_3_ to remove kaolin suspensions and wastewater treatment. The results indicated that the addition of EPS-160 in aggregation system not only largely improved the flocculation ability than the individual use of chemical flocculant (over 30 percent), but also overcome the decrease of flocculation activity due to the overdose of AlCl_3_ and maintained the optimum dosage of AlCl_3_ in a wide range (11–23 mg/L). The zeta potentials and EPS-160 structure indicated that both charge neutralization and bridging were the flocculation mechanism with kaolin. During the wastewater treatment, this composite flocculants consisted of EPS-160 and AlCl_3_ also had great performance for turbidity elimination. Moreover, with the properties of high flocculation activity, hyperthermal stability, pH tolerance and non-toxicity, EPS-160 shows great potential applications.

## Introduction

Flocculants are widely applied in various industrial fields because of the efficient removal ability of colloidal particles, including organic material, suspended solids, and heavy metals. Chemical flocculants including inorganic flocculants and synthetic organic flocculants are most commonly used in industrial applications (Lapointe and Barbeau, 2020). Ferric chloride and polyaluminum chloride are the common inorganic flocculants, but currently not widely used due to the high doses needed, low efficiency and pH sensitivity. Compared with inorganic flocculants, synthetic organic flocculants (such as polyacrylamide derivatives and polyethyleneimine) have relatively higher flocculation efficiency. However, these high-molecular-weight polymers still retain in water environment after treatment, which might cause serious environmental and health problems (Salehizadeh et al., 2018). Thereby, there is an urgent need for a biosafe and sustainable flocculant resource.

Microbial flocculants [extracellular polymeric substances (EPS)] are natural-based polymeric flocculants that are released by microbes in the medium during their growth and/or lysis (Freitas et al., 2017; Srivastava et al., 2018). The main components of these bioflocculants are polysaccharides, proteins, nucleic acids, humic substances and lipids, among others. In recent studies, EPS are considered as a potential alternative for chemical flocculants because of the excellent properties related to non-toxicity, biodegradability, and non-secondary pollution (More et al., 2014). Kinds of prokaryotic and eukaryotic microorganisms isolated from water, soil, and activated sludge have been reported to secrete bioflocculants (Rebah et al., 2018; Li et al., 2020). However, bioflocculants have not yet been produced on a large scale because of their weak flocculating activity and high cost. Therefore, the research for microorganisms with higher production, efficient flocculation ability and low cost are still demanded. Furthermore, research on the flocculation mechanism is also needed in order to improve bioflocculant yields and flocculation activity.

The *Raoultella* species consist of *Raoultella terrigena*, *Raoultella planticola*, *Raoultella ornithinolytica*, and *Raoultella eletrica* in the family *Enterobacteriaceae*. *Raoultella* is reclassified as a new genus distinct from *Klebsiella* based on phylogenetic analysis of 16S rRNA sequence and rpoB gene in 2001 (Drancourt et al., 2001). Unlike the conditional pathogen *Klebsiella* species, *Raoultella* species are considered as relatively safe environmental bacteria since they are rarely reported in clinical samples. *Raoultella* strains with potential biotechnological application have been reported with pullulanase and peroxidase production (Hii et al., 2012; Freitas et al., 2017). Recently, in contrast with petroleum-based 2,3-butanediol (2,3-BD) production, the biological production of 2,3-BD by *R. ornithinolytica* is an environmentally friendly method and has received much attention (Kim et al., 2016). To date, few literatures have reported on the production of bioflocculants from *Raoultella* strains.

In the present study, a high flocculant-producing capability strain, *R. ornithinolytica* 160-1 was isolated and identified from contaminated YPD (yeast peptone dextrose) medium. High flocculation activity of EPS-160 was achieved in kaolin suspension with the aid of trivalent cations, e.g., Al^3+^ and Fe^3+^. To optimize the production of EPS-160, various factors affecting bioflocculant production were optimized through response surface methodology (RSM). The characterization of EPS-160 was evaluated, and the mechanism responsible for flocculation was estimated. Then, the application of EPS-160 combined with chemical flocculant AlCl_3_ was analyzed to remove real wastewater.

## Materials and Methods

### Identification of Strain 160-1

Strain *R. ornithinolytica* 160-1 was cultivated at 30°C in 250-mL flasks containing 50 mL fresh YPD medium (glucose 20 g/L, peptone 20 g/L, and yeast extract 10 g/L) for 12 h with 200 rpm shaking. Then, the cells were recovered and genomic DNA of strain 160-1 was extracted using the TIA Namp Bacterial DNA Kit (TianGen, China). The DNA fragment of the partial 16S rRNA sequence was amplified by PCR with primers 27F (5′-AGAGTTTGATCCTGGCTCAG-3′) and 1492R (5′-GGTTACCTTGTTACGACTT-3′). The sequence of PCR product was determined by Sangon Biotech Co., Ltd (Shanghai, China). The obtained 16S rRNA gene sequence of strain 160-1 was compared with the sequences in the NCBI database^1^. Then, the biochemical characteristics of strain 160-1 were identified using an automatic bacterial identification and susceptibility analysis system (MicroScan WalkAway 96 Plus system, Siemens, Germany). Furthermore, matrix-assisted laser desorption/ionization-time of flight mass spectrometry (MALDI-TOF MS) was employed to differentiate *R. ornithinolytica* and *Klebsiella aerogenes* by comparison of the mass spectra (bioMérieux, Marcy l’Etoile, France) (De Jong et al., 2013).

### Flocculating Activity Determination

The flocculation activity was evaluated according to the standard kaolin suspension method (Salehizadeh and Shojaosadati, 2001). In a 100-mL cylinder, one milliliter each of the sample and AlCl_3_ solution were mixed with 48 mL of kaolin solution (4 g/L). After 5 min at room temperature, the upper phase of the mixture was measured at 550 nm. The control sample consisted of no flocculation agent in the cylinder. The flocculation activity was calculated according to the decrease in turbidity and the equation was as follow: Flocculation activity (%) = (*A*-*B*)/*A* × 100%, where *A* and *B* was the absorbance of the control and the sample at 550 nm, respectively. Each sample was tested independently in triplicate.

### Extraction and Purification of EPS-160

*Raoultella ornithinolytica* 160-1 was cultivated in a 500-mL flask containing 100 mL fermentation medium (MgSO_4_⋅7H_2_O 0.6 g/L, KH_2_PO_4_ 1 g/L, K_2_HPO_4_ 1.5 g/L, yeast extract 0.5 g/L, peptone 10 g/L, and sucrose 30 g/L) at 25°C, 150 rpm shaking for 24 h. The flocculating activity of both cell suspension and the supernatant of fermentation broth were evaluated to facilitate the flocculant extraction. The flocculation efficiency of the cell suspension was very low, whereas the supernatant was found to have high flocculation activity. This phenomenon was consistent with the fact the extracellular EPS with high adsorption abilities was mainly secreted in the medium, while the cells of strain had few flocculation activity (More et al., 2014). Therefore, cell-free supernatant was collected by centrifuged for EPS extraction. The extraction process of EPS-160 from the fermentation broth was conducted as follows. After centrifugation at 8,000 × *g* for 20 min, the cell-free supernatant was obtained and then mixed with three-fold volumes of chilled absolute ethanol. The mixture was stored at 4°C overnight and the precipitate was recovered and washed with ethanol for three times. Finally, the crude bioflocculant EPS-160 was obtained by lyophilization.

### Experimental Design for the Optimization of EPS-160 Production

The effects of the medium composition on EPS-160 production were firstly evaluated through the classical one-factor-at-a-time method. The carbon and nitrogen source are the two most important constituents for improving bioflocculant production. Glucose, xylose, maltose, sucrose, lactose, or soluble starch with the initial concentration of 30 g/L was used in carbon source optimization. Also, peptone, urea, KNO_3_, (NH_4_)_2_SO_4_, or soybean with 10 g/L concentration was selected as the candidates of nitrogen sources. The effects of other medium components that might influence bioflocculant production, including MgSO_4_⋅7H_2_O, K_2_HPO_4_, KH_2_PO_4_ and yeast extract, were also determined. Then, RSM with a three-level, three-factor Box-Behnken design (BBD) was used to optimize the bioflocculant yield. After single-factor experiments, the center points and parameters were selected and then 17 trials for three different variables representing sucrose, (NH_4_)_2_SO_4_ and MgSO_4_⋅7H_2_O were designed. The design and statistical analysis of data were conducted using Design-Expert 8.0 software.

### Factors Influencing Flocculation Activity

There are some important factors affecting EPS-160 flocculation activity, including metal ions, EPS dosage, pH and thermal stability. The cations including NaCl, KCl, MgCl_2_, CaCl_2_, CuCl_2_, FeCl_2_, FeCl_3_, and AlCl_3_ (10 g/L) were used as the candidates to test the effect on flocculation activity. The pH values of the kaolin solution were adjusted to 2.0–12.0 with HCl and/or NaOH solution, and then the flocculating activity of each sample was assayed. Thermal stability of EPS-160 was carried out by measuring the retained flocculating activity after heat treatment (40, 60, 80, or 100°C) for 3 h.

### Characteristics of EPS-160 Bioflocculant

The total polysaccharide content of EPS-160 was measured by the phenol-sulfuric acid (H_2_SO_4_) method (Yin et al., 2014). The presence of protein was determined using the Bradford method (Blum et al., 2008). For monosaccharide composition analysis, 20 mg EPS-160 was hydrolyzed with 2 mL of 2 M trifluoroacetic acid (TFA) at 120°C for 4 h. The hydrolyzate was evaluated by high-performance liquid chromatography (HPLC) (Agilent1200, United States) equipped with a ROA-Organic Acid H^+^(8%) column (Phenomenex, United States) (Zhang et al., 2017). Functional groups of EPS-160 were identified by measuring from 400 to 4000 cm^–1^ wavenumbers in a Nicolet 6700 infrared spectrometer (Thermo Fisher, America). Molecular weights (MWs) of EPS-160 were analyzed by a gel permeation chromatography (GPC) system (LC20, Shimadzu, Japan). The system was equipped with a refractive index (RI) detector (RID-20, Shimadzu, Japan) and a TSK G4000PWXL column (Tosoh, Japan). The MWs was calculated according to the weight-averaged molecular weight.

### Zeta Potential Determination

The zeta potentials of solution were assayed by the NanoZ zeta potential analyzer (Malvern, United Kingdom) to characterize the flocculation mechanism.

### Acute Toxicity of EPS-160

The acute oral toxicity of EPS-160 was determined by intragastric administration in mice. Twenty Kunming mice (SPF grand, k_w_: 23.0 ± 1.0 g, half male and female) was grouped into experimental group and control group. In the experimental group, 55.0 g/L was chosen as the highest possible concentration of EPS-160 solution according to the solubility of sample and the gastric capacity of mice. A single intragastric administration was injected to healthy mice that deprived of food for 12 h with 20 mg EPS-160 aqueous solution per gram of body weight. The control was treated with the same volume of 0.9% saline. The body weight and death were monitored continuously over 14 days.

### The Treatment for Real Wastewater

Domestic wastewater was collected from the treatment plant in Hefei, China. EPS-160 and AlCl_3_ were added into the 100 ml wastewater and mixed at high speed (200 rpm) for 10 min and then for low speed (40 rpm) for 5 min. The chemical oxygen demand (COD) was evaluated by dichromate method. Color and turbidity were analyzed by spectrophotometer at 445 and 860 nm, respectively.

### Statistical Analysis

All experiments were repeated for three times and the standard error of the mean was marked as error bars in figures.

## Results and Discussion

### Strain Characterization and Identification

The colonies of strain 160-1 were round, smooth and protruding on YPD agar medium. They had a milk white color with a diameter of 1–2 mm after 24 h aerobic incubation at 30°C. Cells of strain 160-1 were gram-negative, short and rod shaped, non-spore-forming and capsule-forming. Compared with the published 16S rRNA sequences deposited in the GenBank database, the 16S rRNA gene sequences of strain 160-1 (GenBank accession no: MN400079) shared 99.87 and 99.80% identity with that of *R. ornithinolytica* MG01 and *K. aerogenes* NCTC8846, respectively. The characteristics of strain 160-1 were determined in the automatic bacterial identification and susceptibility analysis system through biochemical and physiological comparison (MicroScan WalkAway 96 Plus system). However, the commercial biochemical identification system did not effectively differentiate *Raoultella* species from *Klebsiella* species (data not shown). Then, MALDI-TOF MS was applied by mass spectra data analysis, and strain 160-1 was identified as *R. ornithinolytica* (Supplementary Figure S1) (de Alegria Puig et al., 2019). Therefore, according to the results of the morphological features of colonies and cells, the physiological and biochemical characteristics, and MALDI-TOF MS identification, strain 160-1 was defined as *Raoultella* and named *R. ornithinolytica* 160-1.

### Synchronous Bioflocculant Production With Cell Growth

**FIGURE 1:**
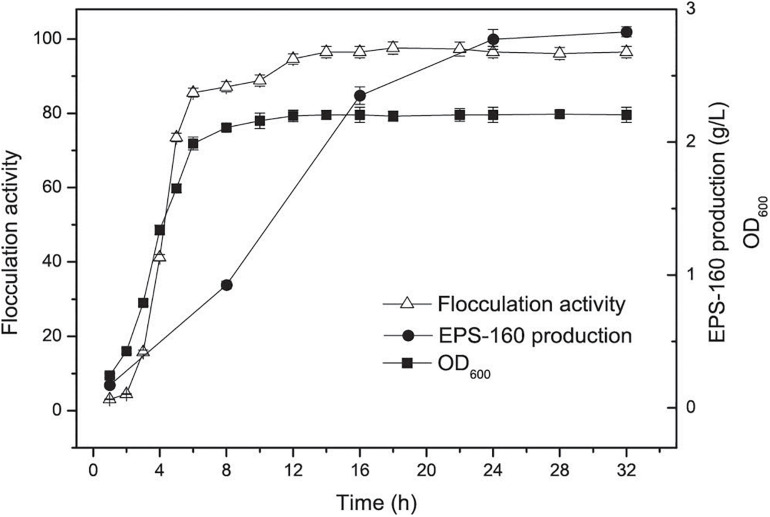
The curves of cell growth, flocculation activity and EPS-160 production during *R. ornithinolytica* 160-1 cultivation.

Bioflocculant EPS-160 production and the growth of *R. ornithinolytica* 160-1 were measured. An increase in both cell growth and flocculation efficiency for *R. ornithinolytica* 160-1 was observed in the first 16 h, and the flocculation efficiency of EPS-160 was synchronous with cell growth (Figure 1). This result indicated that EPS-160 was secreted during growth and differed from some bioflocculants such as MBF-W6 produced by *Chryseobacterium daeguense* W6, which was released by cell autolysis (Liu et al., 2010). Moreover, there was little variation in the flocculating activity of EPS-160 after 18 h. These phenomena indicated that de-flocculation enzymes were not secreted by this strain. *Enterobacter cloacae* degraded the extracellular biopolymer substance when carbon source was exhausted, which was obviously not beneficial for the harvesting and stocking of bioflocculant production (Prasertsan et al., 2006). Moreover, the production of EPS-160 was evaluated at 8, 16, 24, and 32 h, respectively. Although it was not completely consistent with the growth curve, the production of EPS-160 was also synchronous with growth. After a 24-h fermentation, 2.76 ± 0.24 g/L EPS-160 was produced and there was little variation in the flocculant production. Thus, 24 h was selected as the optimal culture time for EPS-160 harvest.

### Improvement of EPS-160 Production by RSM

**FIGURE 2:**
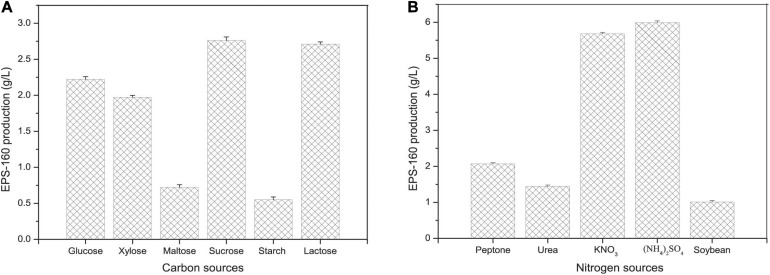
The effect of carbon **(A)** and nitrogen **(B)** sources on EPS-160 production.

**FIGURE 3:**
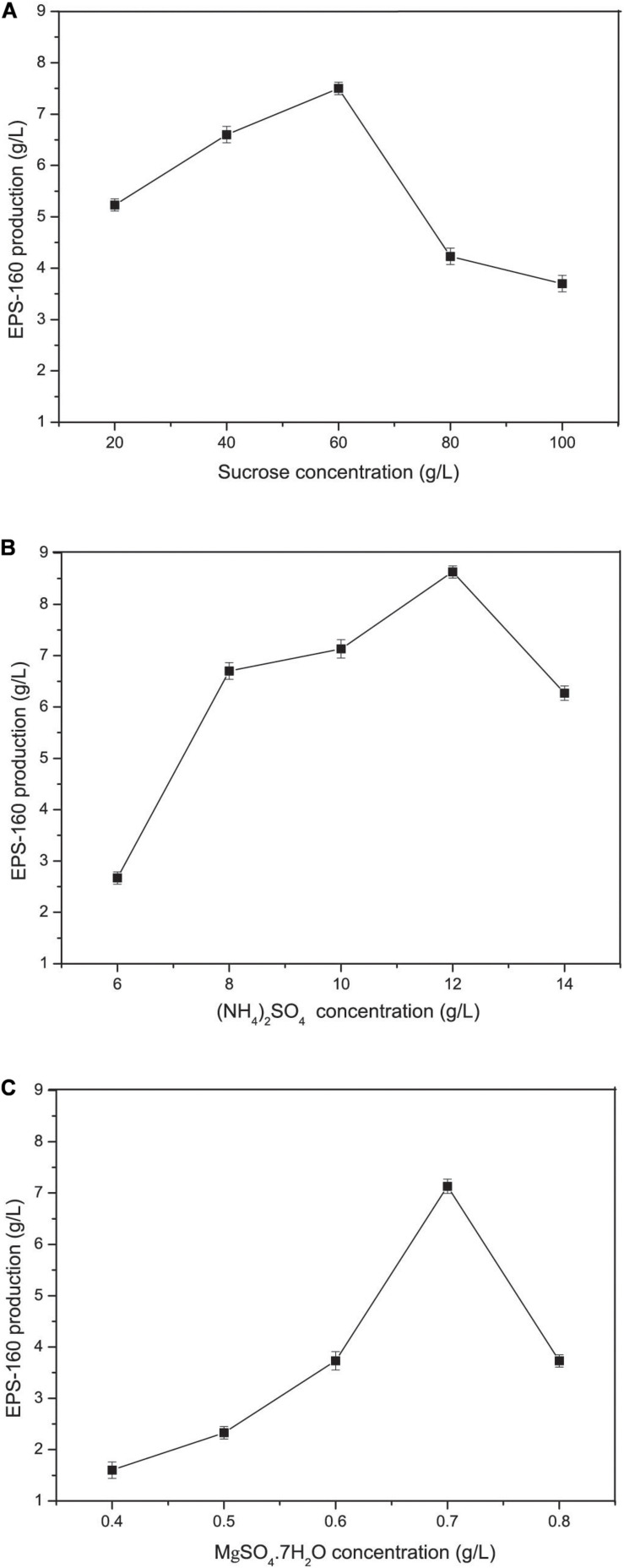
The concentration effects of various medium constituents on EPS-160 production. **(A)** sucrose; **(B)** (NH_4_)_2_SO_4_; **(C)** MgSO_4_⋅7H_2_O.

Carbon and nitrogen sources play key roles in the energy sources for cell growth and important substrates for the synthesis of bioflocculants. The effect of various carbon sources on EPS-160 production was determined, and sucrose was the best candidate for flocculant production (2.74 ± 0.21 g/L) (Figure 2A). Sucrose was the commonly used carbon source in the EPS production since it was consumed easily, available commercially and inexpensive. The optimal concentration of sucrose was 60 g/L with which 7.5 ± 0.14 g/L EPS-160 was produced (Figure 3A). In the tested nitrogen sources, KNO_3_ and (NH_4_)_2_SO_4_ were the preferred nitrogen sources for the production of EPS-160 (5.68 ± 0.11 and 5.99 ± 0.19 g/L, respectively) (Figure 2B). The low cost of (NH_4_)_2_SO_4_ therefore was selected as the nitrogen source for bioflocculant production, and the optimized concentration was 12.5 g/L with maximum production of 8.63 ± 0.19 g/L EPS-160 (Figure 3B). The effect of other medium components was also determined. Figure 3C demonstrated that EPS-160 was improved by the addition of 0.7 g/L of MgSO_4_⋅7H_2_O, but both the lower and higher concentration inhibited its production. Compared with the three factors mentioned above, EPS-160 production was maintained at a level with minor changes in the range of the tested concentration of KH_2_PO_4_ (0.5–1.5 g/L), K_2_HPO_4_ (0.5–2.5 g/L), and yeast extract (0.3–0.7 g/L) (data not shown). Therefore, three key factors (sucrose, (NH_4_)_2_SO_4_ and MgSO_4_⋅7H_2_O) were selected for further analysis with BBD.

Three key factors were selected for RSM, and the variables were 40, 60, and 80 g/L for sucrose; 10, 12, and 14 g/L for (NH_4_)_2_SO_4_; and 0.6, 0.7, and 0.8 g/L for MgSO_4_⋅7H_2_O. The experimental designs in the BBD were shown in Table 1. The relationship between EPS-160 production (*Y*) and the test variables in terms of coded units (*X*_1_, *X*_2_, and *X*_3_) was expressed as follow:

Y=9.970+1.68X1+0.43X2+0.42X3+0.31X1X-20.58X1X-30.11X2X-32.66X1-20.60X22-0.51X32

**TABLE 1:** The matrix of the BBD experiment for culture condition optimization and the corresponding experimental data.

Run	*X*_1_ (Sucrose)		*X*_2_ ((NH_4_)_2_SO_4_)		*X*_3_ (MgSO_4_.7H_2_O)		EPS-160 yield
	Coded	Real level	Coded	Real level	Coded	Real level	(g/L)
	level	(g/L)	level	(g/L)	level	(g/L)	
1	1	80	1	14	0	0.7	9.03
2	0	60	0	12	0	0.7	9.67
3	0	60	1	14	1	0.8	9.32
4	−1	40	0	12	−1	0.6	4.03
5	0	60	0	12	0	0.7	9.42
6	1	80	0	12	1	0.8	7.87
7	0	60	0	12	0	0.7	9.76
8	0	60	−1	10	1	0.8	8.57
9	−1	40	1	14	0	0.7	4.58
10	0	60	−1	10	−1	0.6	7.63
11	0	60	1	14	−1	0.6	8.83
12	−1	40	0	12	1	0.8	6.13
13	1	80	0	12	−1	0.6	8.07
14	0	60	0	12	0	0.7	9.79
15	0	60	0	12	0	0.7	9.88
16	1	80	−1	10	0	0.7	7.67
17	−1	40	−1	10	0	0.7	4.47

where *Y* represented EPS-160 production (g/L) and *X*_1_, *X*_2_, and *X*_3_ were the concentrations of sucrose, (NH_4_)_2_SO_4_ and MgSO_4_⋅7H_2_O.

**FIGURE 4:**
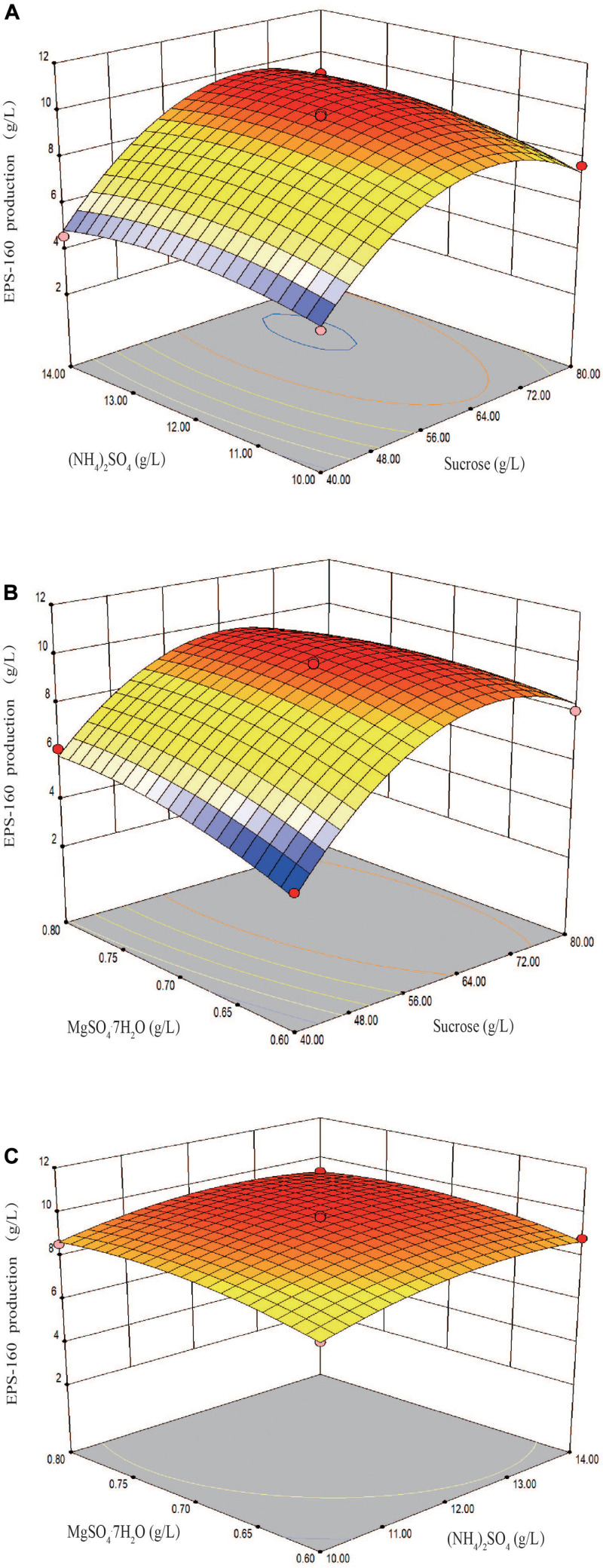
Three-dimension response surface plots of the effect of three variables in medium on EPS-160 production. **(A)** Interaction of sucrose (*X*_1_, g/L) and (NH_4_)_2_SO_4_ (*X*_2_, g/L); **(B)** interaction of sucrose (*X*_1_, g/L) and MgSO_4_⋅7H_2_O (*X*_3_, g/L); **(C)** interaction of (NH_4_)_2_SO_4_ (*X*_2_, g/L) and MgSO_4_⋅7H_2_O (*X*_3_, g/L).

The fit of the regression equation was reliable according to the results in Table 2. The determination coefficient *R*^2^ value was 0.9901, which indicated that 99 percent of the variance in fermentation could be explained by the model. The parameters including *F* value (77.45), *p*-value (<0.0001), and coefficient of variation (CV) (3.75%) indicated that the model was credible. In this case, the linear coefficients (*X*_1_, *X*_2_, and *X*_3_), quadratic term coefficients (*X*_1_^2^, *X*_2_^2^, and *X*_3_^2^) and other term coefficient (*X*_1_*X*_3_) played important roles in bioflocculant production (Table 2 and Figure 4). Based on the canonical correlation analysis, the optimal conditions of sucrose, (NH_4_)_2_SO_4_ and MgSO_4_⋅7H_2_O were 66.41 g/L, 12.84 g/L, and 0.75 g/L, respectively. Under this theoretical fermentation conditions, the maximum EPS-160 output was expected to be 10.19 g/L.

**TABLE 2:** Variance analysis (ANOVA) of the response surface quadratic model for culture conditions of EPS-160 production.

Source	Sum of	df	Mean	*F* Value	*p*-value
	Squares		Square		Prob.*F*
Model	61.43	9	6.83	77.45	< 0.0001^a^
X_1_	22.55	1	22.55	255.85	< 0.0001^a^
X_2_	1.46	1	1.46	16.59	0.0047^a^
X_3_	1.39	1	1.39	15.73	0.0054^a^
X_1_x_2_	0.39	1	0.39	4.43	0.0733
X_1_x_3_	1.32	1	1.32	15.01	0.0061^a^
X_2_x_3_	0.051	1	0.051	0.57	0.4732
X_1_^2^	29.89	1	29.89	339.23	< 0.0001^a^
X_2_^2^	1.53	1	1.53	17.32	0.0042^a^
X_3_^2^	1.11	1	1.11	12.65	0.0093^a^
Residual	0.62	7	0.088		
Lack of fit	0.49	3	0.16	5.34	0.0697
Pure error	0.12	4	0.031		
Cor total	62.04	16			

**R*^2^ = 0.9901; Adj *R*^2^ = 0.9773; CV = 3.75%.*

*df, degrees of freedom.*

*^*a*^Model terms are significant.*

Then, bioflocculant production was examined under the suggested optimal conditions. The actual EPS-160 production secreted by *R. ornithinolytica* 160-1 was 10.01 ± 0.22 g/L after 24 h of fermentation, which was close to the predicted value of the model. Optimal EPS-160 production was approximately 3.63 times greater than under the original non-optimal condition (2.76 g/L), whereas only 2.21 and 1.28 times of carbon and nitrogen source were consumed. Therefore, the unit cost could be reduced regarding the improvement in production efficiency.

Culture time is an important factor for cost control. Only 24 h was used to produce 10.01 g/L EPS-160, which was shorter than the production periods used for previously reported bioflocculants. Low production of bioflocculant producers in *Klebsiella* have been documented: 0.973 g/L for 5 days [*Klebsiella*. sp. S11] (Dermlim et al., 1999), 3.0 g/L for 3 days [*Klebsiella pneumoniae* H12] (Nakata and Kurane, 1999), 2.58 g/L for 9 days [*Klebsiella mobilis*] (Wang et al., 2007), 2.84 g/L for 2 days [*K. pneumoniae* MBF-5] (Zhao et al., 2013), and 240 mg/L for 1.5 days [*Klebsiella oxytoca* GS-4-08] (Yu et al., 2016). Nie et al. (2011) reported the highest yield of MNXY1 (14.9 g/L) produced by *K. pneumoniae* NY1 using dextrin as the carbon source after 3 days of cultivation, with a flocculation activity of 69.17%. In the case of *Klebsiella* sp. PHRC 1.001, high exopolysaccharide production with excellent emulsifying properties was 21 g/L in a 7 L fermentor for 3 days (Zhao et al., 2017). Additionally, the production time was generally between 2 and 4 days in the handful of other genus strains with hyperproduction of bioflocculants. These strains included *Paenibacillus elgii* B69 (25.63 g/L for 4 days) (Li et al., 2013), *Agrobacterium* sp. M-503 (14.9 g/L for 3 days) (Li et al., 2010) and *Nannocystis* sp. NU-2 (14.8 g/L for 2 days) (Zhang et al., 2002). Therefore, strain 160-1 with high productivity (10.01 g/L⋅d) and high flocculation activity (98.33%) is of great prospect in industrial applications with the advantage of reduced cost.

### Characterization of EPS-160

**FIGURE 5:**
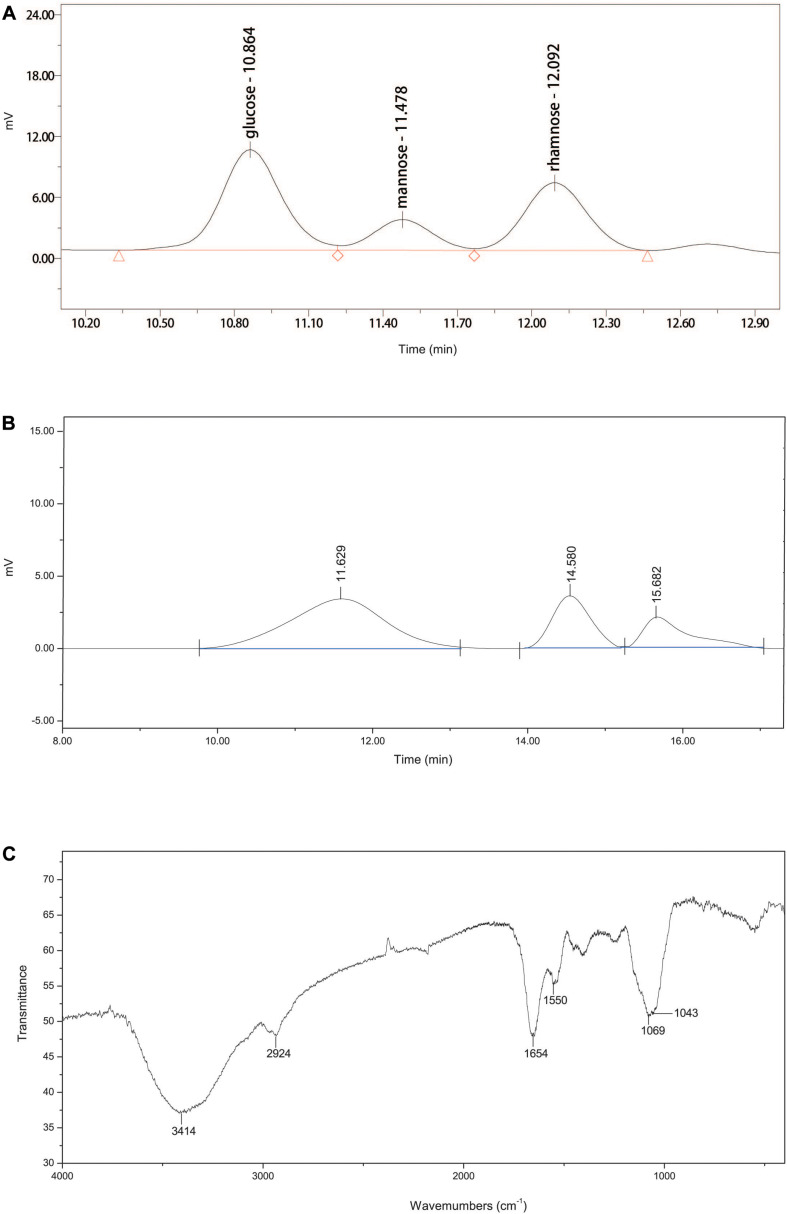
**(A)** The monosaccharide composition of EPS-160. **(B)** The GPC analysis of EPS-160. **(C)** Infrared spectra of EPS-160.

The ultraviolet spectrophotometry results showed no characteristic absorption of nucleic acids at 260 nm, suggesting that EPS-160 contained no nucleic acids (Li et al., 2013). The data from phenol-sulfuric acid method and the Bradford reaction confirmed 80.3% sugar and 5.7% protein, indicating that EPS-160 was mainly composed of polysaccharides. These results agreed with the published reports that the dominant components of EPS were carbohydrates and protein with 75–90% percent (More et al., 2014; Yin et al., 2014; Zhao et al., 2017; Guo et al., 2018). EPS-160 was hydrolyzed with TFA to measure the monosaccharide composition. The HPLC results showed that EPS-160 was comprised of glucose, mannose, and rhamnose at a ratio of 3.1:1:2.4, respectively (Figure 5A). The GPC results showed that three peaks were identified by three retention times (11.629, 14.580, and 15.682 min). The corresponding weight-averaged MWs of the three constituents were 1.17 × 10^6^, 1.12 × 10^4^, and 1.72 × 10^3^ Da, respectively, accounting for a peak area of 69.0, 19.4, and 11.5% (Figure 5B). The infrared spectrometer (IR) spectrum was used for the identification of characteristic organic groups in EPS-160 (Figure 5C). The strong stretching peak at 3414 cm^–1^ validated the presence of large numbers of hydroxyl group, which might be the reason of −OH or −NH vibration in polysaccharide sugar ring (Guo et al., 2014). The weak peak at 2924 cm^–1^ could be attributed to C-H stretching band as the typical of carbohydrates. The strong absorption peak at 1654 cm^–1^ and a weak peak at 1403 cm^–1^ indicated that EPS-160 contained many carboxyl groups (Zheng et al., 2008). The peak at 1550 cm^–1^ showed the existence of NH bending vibrations (Yin et al., 2014). The presence of methoxyl groups could be hinted with the strong stretching peak at 1069 cm^–1^ (Sun et al., 2015). Therefore, EPS-160 contained extensive hydroxyl, carboxyl, and methoxyl groups by the IR spectrum analysis, and this structure could be helpful in the flocculation process against kaolin suspension.

### Flocculating Activity

**FIGURE 6:**
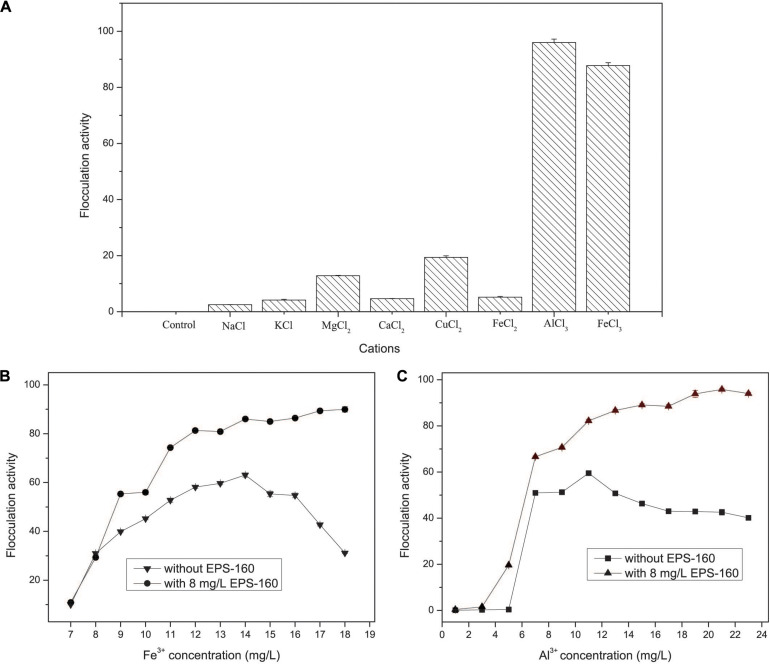
The effects of cations **(A)** and metal concentration **(B,C)** on the flocculation activity of EPS-160 in kaolin suspension.

The flocculation activity of EPS-160 was strongly dependent on metal cations. There was negligible flocculation activity when EPS-160 was present alone, and weak flocculation of EPS-160 was observed with univalent and divalent cations, including Na^+^, K^+^, Mg^2+^, Ca^2+^, Cu^2+^, and Fe^2+^. But when the trivalent FeCl_3_ or AlCl_3_ solution was added in the system, the flocculation was significantly improved, with 87.73 ± 0.74% and 95.86 ± 1.21% turbidity elimination (Figure 6A). The effects of trivalent cation on bioflocculants from other microorganisms were also reported. With the aid of cations (K^+^, Ca^2+^, Mn^2+^, Ba^2+^, Fe^3+^, and Al^3+^), the flocculating activity of EPS secreted by *Micrococcus* sp. Leo occurred and the highest flocculating activity (85.2%) was observed with Al^3+^ (Okaiyeto et al., 2013). Also, the EPS produced by *Nannocystis* sp. NU-2 needed Fe^3+^ and Al^3+^ when it was used in coagulation-flocculation (Zhang et al., 2002).

As we known, FeCl_3_ and AlCl_3_ were the typical inorganic flocculant. Thus, the investigation of the inorganic flocculants and the combined applications of chemical coagulants and EPS-160 were carried out. The flocculant ability of composite bioflocculant, which were combined by EPS-160 and FeCl_3_/AlCl_3_, was higher than chemical flocculants alone (Figures 6B,C). As shown in Figure 5B, the optimal concentration of FeCl_3_ was determined at 14 mg/L with 63.07 ± 1.14% flocculation ability. When the Fe^3+^ concentration was further improved, however, the flocculation rate clearly decreased. This phenomenon possibly attributed to the surface charge reversal of kaolin particles from negative to positive due to Fe^3+^ high charge density. Overdose of a positive charge between suspended particles might reduce flocculation by electrostatic repulsion forces (Salehizadeh et al., 2018). However, when the composite bioflocculant was applied into the kaolin solution, the ability of turbidity decrease was obviously stimulated by 23.02% flocculation activity. Moreover, the flocculation ability of composite bioflocculant was not reduced with the increase concentration of FeCl_3_, and the highest flocculation activity was 89.37 ± 0.66% at 17 mg/L FeCl_3_. That is to say, the addition of 8 mg/L EPS-160 not only strengthened the ability of turbidity elimination for the chemical flocculant, but also overcome the decrease of flocculation activity due to the overdose of FeCl_3_ and maintained the optimum dosage of FeCl_3_ over a wide range (14–18 mg/L). Thus, EPS-160 would be a great candidate as the constituter of the composite bioflocculant in the water treatment for turbidity removal.

The effect of AlCl_3_ and the combination of AlCl_3_ and EPS-160 was also tested in Figure 6C. Similarly, the flocculation activity of composite bioflocculant was higher than the chemical flocculant, with the best flocculation ability of 95.81 ± 0.74%. When the chemical flocculant was used alone, 59.49 ± 0.52% flocculation activity was observed and the optimal concentration of AlCl_3_ was 11 mg/L. However, 82.16 ± 0.69% flocculation ability was easily obtained with the addition of 8 mg/L EPS-160 at this AlCl_3_ concentration. Then, the ability of flocculation increased with the higher concentration of AlCl_3_ and the highest point was established at 21 mg/L with 95.81 ± 0.74% flocculation ability. The effect of the treatment was optimal when the combination proportion of EPS-160 and AlCl_3_ was 8:21. Finally, AlCl_3_ was chosen as the optimal chemical flocculant with EPS-160 for next flocculation assay.

**FIGURE 7:**
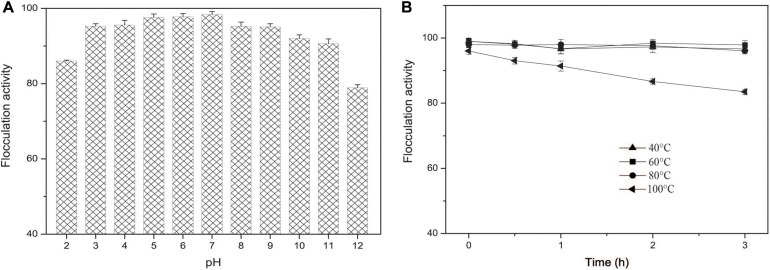
**(A)** The pH effect on the flocculation activity of EPS-160. **(B)** The thermal stability of EPS-160.

The flocculation activity of EPS-160 in a wider pH range from 3.0 to 11.0 was tested and the results indicated that EPS-160 was relatively stable and high efficiency (over 90%) (Figure 7A). Moreover, the thermo-stability of EPS-160 showed more than 80% flocculating activity maintained even after 100°C treatment for 3 h (Figure 7B). In the acute oral toxicity test using Kunming mice, the maximum tested dosage of EPS-160 was 1.1 g/kg body weight. Based on the properties, EPS-160 showed potential application as a safety, stable and high efficiency bioflocculant used in wide pH range.

### Flocculation Mechanism

The zeta potential and structure of EPS-160 suggested that its flocculation process with kaolin combined charge neutralization and bridging mechanism. Charge neutralization, bridging or a combination of them is the main flocculation mechanism reported so far (Salehizadeh et al., 2018). Charge neutralization happens when bioflocculants with opposite charges are added in colloid solution. In this case, polysaccharide flocculants neutralize the oppositely charge particles, and thus the repulsion between particles is eliminated. The zeta potentials of the EPS-160 and kaolin surface were charged with −15.4 and −29.2 mV, respectively. With the addition of EPS-160 or AlCl_3_ into the kaolin solution, the zeta potentials of the EPS-160/kaolin and AlCl_3_/kaolin solution changed to −28.1 and 24.7 mV, respectively. However, the coagulation-flocculation phenomenon did not occur. The observed particles were obviously adsorbed to form flocs when both EPS-160 and AlCl_3_ with optimal concentration were added to the kaolin solution, with a zeta potential of −1.94 mV. Similarly, the negative charge was changed with the addition of EPS-160 (8 mg/L) and FeCl_3_ (14 mg/L) to the kaolin solution (−1.89 mV). These results suggested that the addition of Al^3+^ or Fe^3+^ in this flocculation system was indispensable, and that charge neutralization was one of the flocculation mechanisms for EPS-160. However, this phenomenon could not explain the roles of the other tested univalent and divalent cations in flocculation (Figure 6A), which were normally used as the cofactor to reduce the negative charge on both bioflocculant and kaolin particles and eventually improved coagulation-flocculation efficiency.

A bridging mechanism is another important flocculation mechanism that refers to the assembly of chain-shaped polymers with the colloidal particles or suspended matter through the active site (Salehizadeh and Shojaosadati, 2001). This mechanism depends on the structure of the bioflocculant, including the chemical composition, molecular weight and functional groups. In this study, efficient flocculation did not occur with Na^+^, K^+^, Mg^2+^, Ca^2+^, Cu^2+^, and Fe^2+^. Additionally, the flocculation efficiency of EPS-160 did not drop with the increased concentration of chemical flocculant (Figures 6B,C), which would further reduce the negative charge of the kaolin suspension. These results indicated that bridging mechanism might also be responsible for the coagulation flocculation of EPS-160.

In another study, the flocculation mechanism of Fe^3+^ and Al^3+^-dependent polysaccharides (P-GS408) produced by *K. oxytoca* GS-4-08 was proposed with complex mechanism including charge neutrality, bridging and net catching. The main backbone of P-GS408 comprises galactose and rhamnose with the structure of extensive hydroxyl, amino groups and carboxyl (Yu et al., 2016). Similarly, the high molecular weight of EPS-160 with the presence of functional groups (hydroxyl, carboxyl, and methoxyl) was probably another important reason for the high flocculation rate.

### The Treatment for Real Wastewater

**FIGURE 8:**
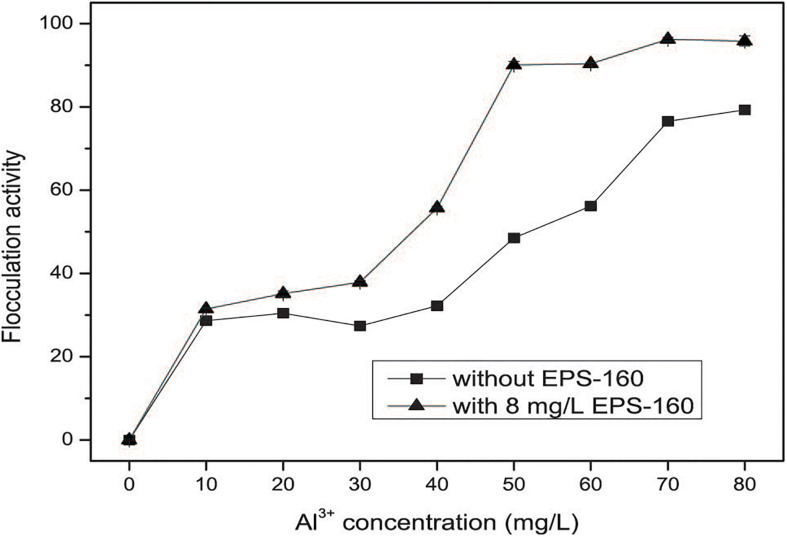
The flocculation activity of EPS-160 coupled with AlCl_3_ in wastewater treatment.

The application of composite bioflocculant combined with EPS-160 and AlCl_3_ could achieve high pollutant removal efficiency when they were used in real wastewater. As shown in Figure 8, the optimum dosage of the coagulants was 80 mg/L when the individual use of AlCl_3_ was taken, with 78.15 ± 0.57% efficiency of turbidity elimination. The combined applications of EPS-160 and AlCl_3_ improved the efficiency and stability of coagulation, and 91.05 ± 1.08% flocculation activity was obtained when they was used at the combination proportion of EPS-160 (8 mg/L) and AlCl_3_ (50 mg/L) for 4:25. Also the pollutant removal efficiencies for COD and color were 74 and 88%, respectively. This result was similar with other bioflocculant coupled with inorganic flocculants in water treatment (Nie et al., 2008). In that report, the turbidity efficiency of bioflocculant from bacteria NII4 could be obviously reinforced when FeCl_3_ or AlCl_3_ was added in suspended solid solution. Since the negative impact of aluminum salts with high dosage to environment and human health, the exploring of composite flocculants with bioflocculant coupled with inorganic salts is very promising. Chao et al. (2020) reported a novel coagulant carboxymethypullulan-AlCl_3_ with enhanced flocculation activity and decreased dosage of AlCl_3_ in kaolin suspension. In this study, the substantial reduction use of AlCl_3_ and high removal rate indicated that the composite flocculant prepared from EPS-160 and AlCl_3_ had great potential in real wastewater treatment.

## Conclusion

In this work, an efficient bioflocculant-producing strain, *R. ornithinolytica* 160-1, was isolated. This strain produced a novel Al^3+^-dependent, highly efficient, thermally stable and non-toxic bioflocculant, EPS-160. A maximum EPS-160 production of 10.01 g/(L⋅d) was achieved under optimized conditions. EPS-160 achieved great performance for turbidity elimination for the treatment of kaolin suspension and real wastewater when used in combination with inorganic flocculant AlCl_3_. Charge neutralization and bridging mechanism was proposed as the main mechanism for flocculation process. With the excellent properties of high production and efficient flocculation activity for this bio-based flocculant, the feasibility of EPS-160 in industrial application is promising.

## Data Availability Statement

The datasets presented in this study can be found in online repositories. The names of the repository/repositories and accession number(s) can be found in the article/Supplementary Material.

## Ethics Statement

The animal study was reviewed and approved by Experimental Animal Ethics Committee of Anhui Medical University.

## Author Contributions

RD, LL, RH, and MZ performed the experiments and analyzed the data. TL and JT contributed for material preparation. RD and LL designed the experiments. RD wrote the manuscript. SH and JH revised the manuscript. All authors contributed to the article and approved the final manuscript.

## Conflict of Interest

The authors declare that the research was conducted in the absence of any commercial or financial relationships that could be construed as a potential conflict of interest.

## Footnotes

1 http://blast.ncbi.nlm.nih.gov/Blast.cgi

## Supplementary Material

Supplementary Figure 1 Matrix-assisted laser desorption/ionization-time of flight mass spectrometry of strain160-1.
