# Supplementary figures and images for: Rapid Production of a Novel Al(III) Dependent Bioflocculant Isolated From Raoultella ornithinolytica 160-1 and Its Application Combined With Inorganic Salts
Source: Front Microbiol. 2021 Jan 12;11:622365. doi: 10.3389/fmicb.2020.622365 (PMC7835285; doi:10.3389/fmicb.2020.622365)

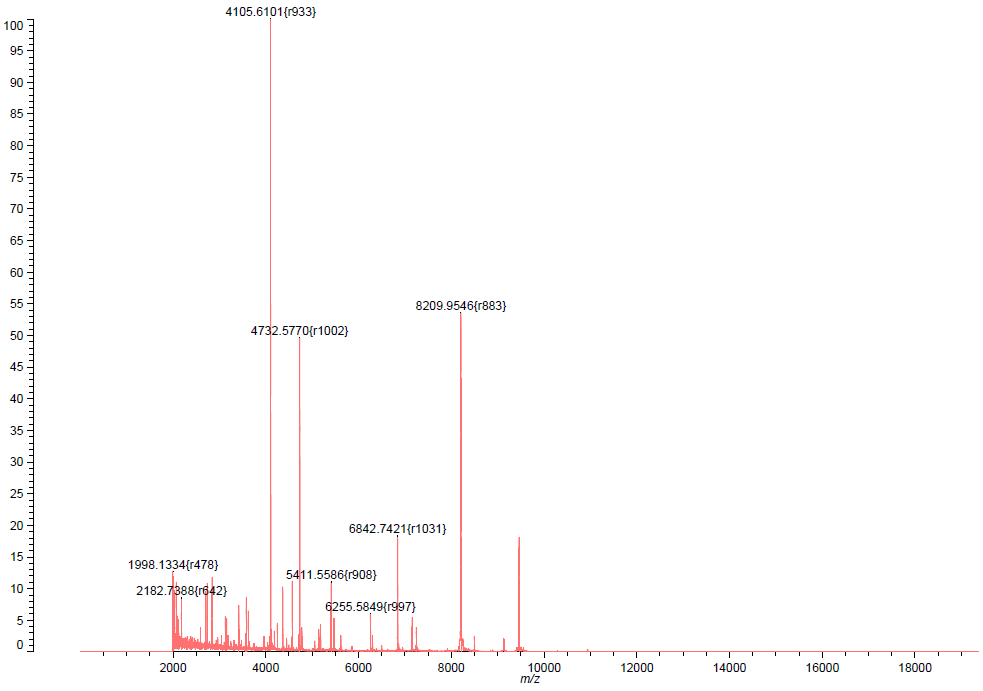

Supplement: Supplementary Figure 1 — Matrix-assisted laser desorption/ionization-time of flight mass spectrometry of strain160-1. [file Image_1.tif]
